# Body Image Evaluation and Sociocultural Attitudes Toward One’s Own Body Among Women Practicing Pole Dance

**DOI:** 10.3390/nu18050814

**Published:** 2026-03-02

**Authors:** Wiktoria Staśkiewicz-Bartecka, Julia Lubojańska, Aleksandra Kołodziejczyk, Agata Kiciak, Agnieszka Białek-Dratwa, Marek Kardas

**Affiliations:** 1 Department of Food Technology and Quality Evaluation, Department of Dietetics, Faculty of Public Health in Bytom, Medical University of Silesia in Katowice, ul. Jordana 19, 41-808 Zabrze, Poland; julia.lubojanska@gmail.com (J.L.); d201294@365.sum.edu.pl (A.K.); akiciak@sum.edu.pl (A.K.); mkardas@sum.edu.pl (M.K.); 2 Department of Human Nutrition, Department of Dietetics, Faculty of Health Sciences in Bytom, Medical University of Silesia in Katowice, ul. Jordana 19, 41-808 Zabrze, Poland; abialek@sum.edu.pl

**Keywords:** pole dance, body image, self-esteem, BES, SATAQ 3, internalization of the ideal of beauty, body in sport

## Abstract

**Background/Objectives:** Sociocultural attitudes toward appearance and body image are important components of women’s psychological well-being, particularly in the context of physical activities involving body exposure, such as pole dance. The aim of this cross-sectional study was to compare body image and sociocultural attitudes toward appearance between women practicing pole dance and women not engaged in this activity, and to examine the associations between these variables. **Methods:** The study included 207 women practicing pole dance (PDG) in clubs and schools across Poland and 180 women not practicing this discipline, who served as the control group (CG). Data were collected using the CAWI (Computer-Assisted Web Interview) method with a proprietary questionnaire and standardized tools: the Sociocultural Attitudes Towards Appearance Questionnaire 3 (SATAQ 3) and the Body Esteem Scale (BES). **Results:** Women practicing pole dance had lower mean BMI and were less frequently overweight but more frequently underweight compared to the control group. They obtained significantly higher scores on the Internalization–Pressure and Internalization–Athlete scales of the SATAQ 3. Significant between-group differences in body image were observed only for the Physical Condition subscale of the BES, with higher scores in the pole dance group. Significant negative correlations were identified between sociocultural attitudes toward appearance and body image in both groups, with stronger associations observed among women practicing pole dance. **Conclusions:** Participation in pole dance was associated with higher self-evaluation of physical condition as well as stronger internalization of sociocultural appearance norms. Due to the cross-sectional design, the findings indicate associations rather than causal relationships. The results underline the importance of preventive and educational strategies promoting a functional rather than exclusively esthetic approach to the body.

## 1. Introduction

Physical activity plays a key role in human life, yet its widespread deficiency is one of the main public health challenges of the 21st century [1,2]. The low level of implementation of activity recommendations highlighting the importance of alternative and attractive forms of exercise that encourage activity among people of all ages [1,3,4].

Pole dancing is an increasingly popular form of physical activity, combining elements of dance, gymnastics and acrobatics, requiring strength, precision and technique [5,6,7]. It is a form of artistic expression that allows for the expression of emotions through choreography. It also brings psychological benefits, such as increased self-confidence and a sense of belonging to a supportive community associated with this form of activity [5,6,7,8,9]. Due to the combination of physical exercise and movement expression, it can provide a specific context for developing body awareness and focusing on functional aspects of body, which can influence body image [7,8,9,10,11].

Body image is a multidimensional construct encompassing numerous interrelated elements [12]. Its perception is influenced by social and cultural factors, including family, peers, and media norms [13,14,15,16,17,18]. Its perception is individual and dynamically changes depending on an individual’s social situation. A positive body image associated with maintaining health and mental well-being and is associated with higher self-esteem, while disturbances in this area can are associated with a higher risk of dysfunctional social relationships or eating disorders [19]. Nowadays, media are widely used in social life [16]. Internalization of the patterns promoted within them can lead to dissatisfaction with one’s appearance [17,19]. At the same time, the positive impact of body positivity, body neutrality, and femvertising movements, promoting body diversity, acceptance, and functionality, has been observed [20,21,22].

Previous research indicates a significant impact of physical activity on body image [9]. However, there is limited empirical research on the relationship between pole dancing participation and body image and sociocultural attitudes towards appearance. This study fills this gap by providing a comparative analysis of women practicing pole dancing and women not participating in this activity, and by examining the relationships between body image and sociocultural attitudes. The hypothesis was formulated that women practicing pole dancing would have a higher self-assessment of their body image compared to women in the control group (CG). It was also assumed that pole dancing participants would demonstrate different in sociocultural attitudes towards body image compared to women who do not engage in this activity. Furthermore, it was assumed that there is a relationship between body perception and sociocultural attitudes towards body image in both research groups.

The present study was conducted in Poland, where pole dance has gained substantial popularity in recent years, both as a recreational activity and as a developing competitive discipline. Despite this growth, research on the psychological aspects of pole dance participation in the Polish population remains limited. Moreover, Poland represents a sociocultural context characterized by increasing exposure to globalized media content and evolving beauty standards, which may influence body image and the internalization of appearance ideals among women. The availability of validated Polish versions of the SATAQ-3 and BES further supported the selection of this study locale.

## 2. Materials and Methods

### 2.1. Procedure for the Study

The study was conducted from May 2024 to March 2025. It included women who practiced pole dance in clubs and schools across Poland, as well as women who did not practice the sport, who served as a CG. A non-probability purposive sampling strategy with elements of convenience sampling was applied. Participants were recruited through social media platforms and online forums dedicated to pole dance, as well as through general social media channels in the case of the control group. This approach enabled the inclusion of women with specific experience related to pole dance practice while also recruiting women from the general population for comparison. Participation in the study was voluntary and anonymous, and no financial or material incentives were offered.

Data were collected using the CAWI (Computer-Assisted Web Interview) method using a questionnaire delivered via the Google Forms platform. The structured online questionnaire was accessed via a dedicated URL link. The questionnaire for PDG was distributed on social media and forums dedicated to the discipline. For the CG, the questionnaire was distributed similarly via social media.

Before data collection, participants were informed of the study topic, its purpose, and anonymity. This information was presented on the first page of the questionnaire. This study was conducted in accordance with the Declaration of Helsinki of the World Medical Association. This study received approval from the Bioethics Committee of the Medical University of Silesia in Katowice (approval code: BNW/NWN/0043-3/641/35/23; approval date: 22 September 2023).

Due to the non-random recruitment strategy and online distribution of the questionnaire, the study may be subject to self-selection bias, and the findings should be interpreted with appropriate caution.

### 2.2. Participants

The study involved 387 participants. The PDG included 207 women aged 28.52 ± 6.37 years. Inclusion criteria for the study group included: (1) female gender, (2) 16 years of age or older, (3) participation in classes at the schools and clubs included in the study, (4) pole dance training for at least 3 months, (5) internet access—social media or groups dedicated to pole dance—and (6) consent to participate in the study. Exclusion criteria included: (1) male gender, (2) age under 16 years, (3) pole dancing for less than 3 months, and (4) incorrectly or incompletely completed questionnaires.

The CG consisted of 180 women who did not practice pole dance. Inclusion criteria for the CG included: (1) female gender, (2) age 16 or older, (3) not participating in pole dancing classes, (4) internet access, (5) consent to participate in the study. Exclusion criteria included: (1) male gender, (2) age under 16, and (3) incorrectly or incompletely completed survey. The CG included women with varying levels of physical activity, including both inactive individuals and women declaring regular activity consistent with current health recommendations [23,24]. This approach allowed for a comparison of pole dancing women with the general population, rather than solely with physically inactive individuals.

Based on these exclusion criteria, three participants were excluded from the study.

### 2.3. Survey Tools

The study utilized a proprietary survey questionnaire to collect metric data and standardized research tools, including the Sociocultural Attitudes Towards Appearance Questionnaire 3 (SATAQ 3) and the Body Esteem Scale (BES).

The proprietary questionnaire varied depending on the group, allowing for adaptation to the specific experiences of the participants. For both groups, data were collected on age, height, current and expected body weight, place of residence, level of education, and the elimination of specific food products from the diet.

In the PDG, the questionnaire was expanded to include questions regarding pole dance training experience, the type of pole dance practiced, and the number of training sessions per week. The question regarding the number of training sessions was open-ended and was excluded from further analysis due to some respondents considering other forms of physical activity, which could have led to ambiguous data interpretation.

In the CG, a question regarding the amount of time engaged in physical activity per week was included. In the case of declaring at least 150 min of moderate intensity activity or 75 min of high intensity activity per week, respondents provided additional information on the most frequently undertaken physical activity, training experience and the number of training units per week.

#### 2.3.1. Nutritional Status

The body mass index (BMI) of the study participants was calculated using the following formula:

$$BMI=\frac{body\;weight(kg)}{{height(m)}^{2}}$$

Data regarding body weight and height were obtained from questionnaires completed by the participants. The results were interpreted according to the WHO classification [25], presented in Table 1.

#### 2.3.2. SATAQ 3

The Polish, validated version of SATAQ 3 was used to assess the impact of sociocultural norms promoted by mass media on attitudes and behaviors regarding the body and physical appearance among the surveyed women [26,27]. The questionnaire assesses the extent to which patterns promoted in mass media, such as magazines, television, and the internet, are taken into account in assessing one’s body image and appearance. This tool can be used with adults and adolescents aged 12 and over [27].

The SATAQ 3 consists of 28 items. The survey involves the participant’s subjective assessment of their opinion on the role of sociocultural standards in shaping attitudes toward their body and appearance. Each item is rated on a five-point Likert scale, where “1” indicates “strongly disagree”, “and “5” means “strongly agree” [27].

The SATAQ 3 includes four scales. The Internalization-Pressure scale comprises 12 items and measures the level of perceived pressure from the media and the degree of internalization of media-promoted appearance standards. High scores on this scale indicate strong compliance with the presented norms. The Information Seeking—Internalization scale comprises 6 items and refers to the active search for information in the media regarding ideal appearance. High scores indicate a strong interest in the promoted standards. The Internalization—Athlete scale comprises 4 items and refers to the perception of an athletic physique as the ideal. A high score indicates the need to strive for a body similar to that of an athlete. The Information scale comprises 6 items and assesses the frequency with which information about appearance is sought in the media. High scores indicate a strong interest in the topic [27].

Table 2 presents the classification of SATAQ 3 questions into individual scales.

Table 3 presents the sten norms of the SATAQ 3 developed for women.

#### 2.3.3. BES

The Polish version of the BES, developed and validated by Lipowska and Lipowski [28,29], was used to assess the self-esteem of study participants. The scale comprises 35 items defining body parts and their functions, and the interpretation of results takes into account the age of the participants. The tool is divided into three subscales, differing by gender. For women, the subscales include Sexual Attractiveness, Weight Concern, and Physical Condition. Sexual Attractiveness refers to the perception of body parts whose appearance cannot be modified through physical activity, but only through cosmetic procedures, e.g., lips and breasts. Weight Concern focuses on body aspects that can be shaped through exercise or dietary changes, e.g., thighs and abdomen. Physical condition reflects the subjective assessment of body fitness, encompassing strength, agility, and endurance [28,29].

Responses to individual items were provided using a five-point Likert scale, where 1 indicates strongly negative feelings, 3 indicates a neutral attitude, and 5 indicates strongly positive feelings [28,29].

Table 4 presents the sten norms for the BES for women.

### 2.4. Statistical Analysis

Statistical analyses were performed using Statistica 13.3 (TIBCO Software Inc., Palo Alto, CA, USA) and LibreOffice Calc 24.8.6.2 (The Document Foundation, Berlin, Germany). Quantitative variables were characterized by minimum (min), maximum (max), mean (X) values, and standard deviations (SD), while qualitative variables were presented as percentages.

The Shapiro–Wilk test was used to verify compliance with a normal distribution. In the absence of compliance with a normal distribution, the significance of differences between the PDG and the CG was verified using the Mann–Whitney U test. The chi-square test of independence was used to compare nominal variables.

For non-parametric comparisons, the effect size was calculated as r. The magnitude of r was interpreted according to Cohen’s criteria (0.10—small, 0.30—medium, 0.50—large).

*p* < 0.05 was used as the criterion for statistical significance.

Given the number of correlation analyses performed, these analyses should be considered exploratory and interpreted with caution.

## 3. Results

### 3.1. Group Characteristics

The study involved 387 women divided into two groups: a group of women practicing pole dancing (PDG) and a control group (CG). Characteristics of both groups are presented in Table 5.

Among all surveyed women, 76.74% (*n* = 297) declared a desire to lose their current body weight. In the PDG, this percentage was 71.50% (*n* = 148), compared to 82.78% (*n* = 149) in the CG. 14.47% (*n* = 56) of participants expressed satisfaction with their current body weight, while 6.72% (*n* = 26) indicated that their expected body weight was higher than their current weight. Statistical analysis revealed significant differences in the expected body weight of the surveyed women between the groups.

BMI analysis revealed that the majority of participants were within the normal range (74.42%, *n* = 288). Underweight was found in 3.88% of women (*n* = 15), overweight in 15.25% (*n* = 59), and obesity in 6.46% (*n* = 25). PDG was characterized by significantly lower body weight and BMI compared to CG. Underweight was more common in PDG (6.26%) than in CG (1.11%). The combined prevalence of excess body weight, including overweight and obesity, was 13.53% (*n* = 28) in the PDG and 31.11% (*n* = 56) in the CG. Statistically significant differences were found in the interpretation of the BMI of the surveyed women between the groups.

The largest percentage of participants had higher education (*n* = 256; 66.15%). Higher education was more common in the PDG (*n* = 147; 71.01%) than in the CG (*n* = 109; 60.56%), while secondary education was more common in the CG (*n* = 58; 32.22%) than in the PDG (*n* = 56; 27.05%). Differences in education level between the groups were statistically significant.

Analysis of place of residence revealed that the highest percentage of participants lived in cities with populations exceeding 250,000 (35.14%, *n* = 136), and the lowest in rural areas (11.89%, *n* = 46). The percentage distribution for rural areas and large cities was similar in both groups, while differences were observed between the groups in cities with populations of up to 100,000 and up to 250,000. No statistically significant differences were found in the place of residence of the surveyed women between the groups.

The distribution of training experience among the women practicing pole dancing is presented in Table 6.

In the PGD (*n* = 207), the largest proportion were women practicing both pole dancing and exotic pole (*n* = 115; 55.56%). Women practicing pole dancing exclusively accounted for 41.06% (*n* = 85), while women practicing exotic pole constituted only 3.38% (*n* = 7).

Women in the CG who engaged in less than 150 min of moderate-intensity activity per week or less than 75 min of vigorous-intensity activity per week constituted 65.00% (*n* = 117) of this group. Women in the CG who met the WHO’s physical activity recommendations, whose physical activity was more than 150 min of moderate-intensity activity per week or more than 75 min of vigorous-intensity activity per week, constituted 35.00% (*n* = 63) of the CG.

Among the CG participants who achieved the recommended activity levels, the most common forms of exercise were strength training (*n* = 20; 31.75%), walking (*n* = 11; 17.46%), running (*n* = 8; 12.70%), and dancing other than pole dancing (*n* = 7; 11.11%). Other activities, such as aerobics/fitness, team games, cycling, yoga/Pilates, Nordic walking, swimming, tennis/table tennis/squash, and others, were less popular, as they were undertaken by a maximum of 4 people.

### 3.2. SATAQ 3

The results of the SATAQ 3 analysis revealed statistically significant differences between groups on the Internalization-Pressure and Internalization-Athlete scales. Women in the PDG achieved significantly higher mean scores and higher sten scores on these scales than participants in the CG. Analysis of the remaining scales revealed no significant differences between the groups (Table 7).

### 3.3. BES

Analysis of the results obtained using the BES revealed statistically significant differences between groups on the Physical Condition subscale. Statistical significance was found only in the mean scores obtained on this subscale. In the remaining subscales of the BES questionnaire, no significant differences were found between the groups (Table 8).

### 3.4. The Correlation Between Body Image and Sociocultural Attitudes Towards Body Image

All statistically significant correlations between the BES totals and the SATAQ 3 scale totals were negative for both the PDG and the CG.

In the Sexual Attractiveness subscale of the PDG, the strongest statistically significant negative correlation was observed with Scale 1 Internalization-Pressure and was of medium strength. Significant correlations were also observed with Scale 2 Internalization—Information Seeking and Scale 4 Information, although their strength was weak. In the CG, negative correlations of medium strength were observed with Scale 1 Internalization-Pressure and Scale 4 Information, while the correlations with Scales 2 and 3 were weak.

Among the correlations between the total score on the Weight Concern subscale and the total score on the SATAQ 3 scales in both the PDG and the CG, the strongest statistically significant negative correlation was found between the total score on the Weight Concern subscale and the total score on Scale 1 (Internalization-Pressure). In the PDG, the correlation was strong, while in the CG, it was medium.

In the Physical Condition subscale of the PDG, significant negative correlations were found with Scale 1 Internalization-Pressure and Scale 4 Information, and their strength was moderate, while the correlation with Scale 2 was weak. In the CG, significant correlations were weak and concerned Scale 1 Internalization-Pressure and Scale 4 Information.

Analysis of the correlation coefficients indicates that a greater number of significant relationships were observed in the PDG than in the CG. The values of the correlation coefficients analyzed in both groups ranged from weak to strong effects, with the highest absolute value of the r coefficient recorded in the PDG for the relationship between Weight Control and Scale 1 Internalization-Pressure. Detailed R coefficient values and significance levels are presented in Table 9.

Analysis of the correlation between the sten scores in the BES and the sten scores on the SATAQ 3 scales revealed a negative direction in all statistically significant correlations in both the PDG and the CG, indicating an inverse relationship between BES scores and SATAQ 3 scores.

In the Sexual Attractiveness subscale of the PDG, statistically significant negative correlations were observed with Scale 1 Internalization-Pressure, with a medium correlation strength, and with Scale 2 Internalization- Information Seeking, with a weak correlation strength. The correlation with Scale 3, Internalization-Athlete, despite a relatively high r coefficient, did not reach statistical significance. In the CG, the strongest negative correlations were observed with Scale 1 Internalization-Pressure and Scale 4 Information. These correlations in the CG were of medium strength.

Among the correlations between the sten scores on the Weight Concern subscale and the sten scores on the SATAQ 3 scales in the PDG, the strongest statistically significant negative correlation was found between the sten scores on the Weight Concern subscale and the sten scores on Scale 1: Internalization-Pressure, Scale 2: Internalization- Information Seeking, and Scale 4: Information, and was of medium strength. In the CG, there was one statistically significant correlation with the sten scores on Scale 1: Internalization-Pressure of the SATAQ 3, and it was weakly negative.

Among the correlations between the sten scores on the Physical Condition subscale and the sten scores on the SATAQ 3 scales in the PDG, the strongest statistically significant negative correlation was found between the sten scores on the Physical Condition subscale and the sten scores on Scale 1: Internalization-Pressure, and it was of medium strength. In the CG, there were two weak negative correlations between the sten scores in the Physical Condition subscale and the sten scores in Scale 1 Internalization-Pressure, and the sten scores in Scale 4 Information.

The Sten analysis revealed that statistically significant negative correlations between self-esteem and sociocultural attitudes toward body image were more frequent in the PDG than in the control group, particularly for the Weight Concern and Physical Condition subscales. For the Sexual Attractiveness subscale, the strength of the correlation was comparable between the two groups. All correlations that reached statistical significance were negative, indicating consistency in the direction of effects between the BES subscales and the SATAQ 3 scales (Table 10).

## 4. Discussion

The assessment of the nutritional status of the surveyed women showed statistically significant differences between women practicing pole dance and women not practicing this discipline. In the CG, excess body weight (including both overweight and obesity) was significantly more common than in women who practiced pole dancing. Underweight was more common in the PGD than in the CG, which may be related to higher energy expenditure due to physical activity or set body goals. It should be noted that data regarding weight and height were self-reported, which may introduce some inaccuracies. Despite this limitation, the results suggest potential effects of regular pole dancing. The results suggest that regular pole dancing may have a beneficial effect on maintaining a healthy body weight, but the presence of underweight among women practicing this discipline may indicate that this group is particularly vulnerable to developing body image disorders. The obtained results partially correspond with the study by Ballarin et al. [30], who demonstrated beneficial changes in body composition in women practicing pole dancing, including a lower percentage of body fat and higher lean mass. However, contrary to their observations, in the present study, significant differences in BMI were observed between the training and control groups, which may be due to different methodological assumptions and sample characteristics. The focus on low body weight, which may accompany appearance-sensitive disciplines, according to the study by Sundgot-Borgen [31], increases the risk of restrictive dietary practices and the development of eating disorders. Increased social and environmental pressure among athletes regarding body shape, described by Martínková et al. [32], may also lead to reduced self-esteem. Consequently, regular engagement in activities with a strong esthetic component may simultaneously promote improvement in body composition parameters and increase susceptibility to risky weight control strategies, justifying the need for preventive measures and monitoring nutritional status in this group.

The hypothesis that women practicing pole dancing exhibit differences in sociocultural attitudes toward body image compared to women in the CG was also partially confirmed, as statistically significant differences were found only on the Internalization-Pressure and Internalization-Athlete scales. Women practicing pole dancing felt media pressure regarding their appearance to a greater extent than women who did not practice pole dancing, and internalized beauty standards presented in the media more strongly. Furthermore, these women were characterized by a higher degree of internalization of norms regarding an athletic physique, i.e., the physique characteristic of someone who regularly and professionally practices sports. The above PDG results suggest that participants identify more closely with the athletic body ideal, which may be due to social norms promoting a slim and athletic appearance, reinforced by social media [33]. Differences between groups may be the result of both physical and cultural aspects of pole dancing. Training develops strength, flexibility, and body awareness, and at the same time, it is an activity with a strong esthetic and performative component, in which figure and appearance play an important role, which favors focusing on the athletic aspect of the body [34]. The results may suggest that the group of physically active individuals is more susceptible to adopting patterns promoted by the media, particularly in terms of an athletic physique and the determination to achieve it. This is confirmed by Nerini’s research on ballet dancers, in whom the internalization of the athletic body ideal was significantly higher than in the group of physically inactive girls [35].

The hypothesis that women practicing pole dancing have a higher self-esteem compared to non-training women was partially confirmed, as statistically significant differences were found only in the Physical Condition scale, where women practicing pole dancing achieved higher mean and sten scores. These results may indicate good self-esteem due to Physical activity. These results are partially consistent with the research by Zhang et al., which showed that physical activity promotes higher self-esteem and satisfaction with one’s own physical condition and strength [36]. In the Sexual Attractiveness subscale, no statistically significant differences between groups were found in any of the questions. In the Weight Concern subscale, statistical significance was found in selected questions, which may indicate that practicing pole dancing determines the perception of certain aspects of physical appearance related to body weight.

The hypothesis regarding the relationship between body image and sociocultural attitudes toward body image was partially confirmed, as not all comparisons demonstrated statistical significance. More statistically significant correlations were found for women practicing pole dancing, while all statistically significant correlations for both groups were negative. The demonstrated correlations indicate that the higher the level of internalization and pressure of sociocultural norms regarding appearance promoted in the media, the lower the self-assessment regarding sexual attractiveness, aspects related to body shape and weight, and physical condition in both the PDG and the CG. It should be noted that the correlation analyses were exploratory in nature, and due to the number of comparisons performed, the findings should be interpreted cautiously and verified in future studies. This pattern is consistent with the research on women and teenage girls by Izydorczyk et al., which demonstrated the significant role of sociocultural predictors in the development of body dissatisfaction and pathological striving for thinness [37].

According to research conducted by the Public Opinion Research Center (CBOS), Poles, primarily women, consider their appearance important. Currently, approximately one-quarter of Poles are dissatisfied with their appearance, and a particular desire to change their appearance is observed among people under the age of 24 [38].

Physically active individuals, including professional athletes, are among groups particularly vulnerable to constant pressure related to socially accepted norms regarding physical appearance. Body image impairment and eating disorders among athletes are difficult to detect. A focus on body shape is often perceived positively, as it positively impacts athletic performance [39,40].

The literature on esthetic sports indicates a risk of body dissatisfaction, particularly in gymnastics and ballet [39,41,42,43,44,45]. In gymnasts, the highest levels of dissatisfaction are observed during the competition period, which may be due to frequent appearances in figure-flattering attire and exposure to judges and social evaluation [41]. Ballet dancers, despite being satisfied with their daily appearance, feel the need to have a slimmer physique during performances [46,47]. Greater experience in sports and dance is associated with more frequent self-observation of one’s own body and a greater internalization of sociocultural norms and norms related to an athletic physique [48].

Esthetic sports also frequently demonstrate a risk of developing eating disorders [39,42,44,45]. Gymnasts competing at an international level practice more restrictive eating, which contributes to anorexia nervosa [39], with the highest severity of symptoms observed during the pre-competition period [41]. In the case of ballet dancers, greater body dissatisfaction was associated with the severity of eating disorders, and dance identity influenced body image expectations [46]. Self-esteem depends on factors such as parental pressure, BMI, and training experience [43]. In elite gymnasts, the main factor contributing to dissatisfaction was poor eating behavior, while in non-elite athletes and untrained individuals, internalization of appearance patterns promoted in the media [41].

Not all forms of physical activity are associated with impaired body image. Budnik-Przybylska’s study, which examined dancers of various styles and non-dancers, showed that dancers perceived their attractiveness and physical fitness as higher, but also focused significantly on their body weight [49]. A study of women participating in recreational pole dancing showed that after 8 weeks of regular training, body satisfaction, physical fitness, strength, flexibility, and coordination improved [50]. These results suggest that recreational physical activity can support self-esteem and a positive body image, in contrast to competitive sports, where the pressure on physique and performance is much greater.

Pole dancing may influence body image through coexisting psychological mechanisms combining functional and esthetic components [9]. Regular pole dancing training promotes improved fitness, strength, flexibility, and coordination, which may increase a sense of physical competence and body satisfaction [34], and mastering new moves may enhance self-efficacy and positively impact self-esteem [51]. According to the review by Liu et al., dancing may provide mental health benefits, promising improved well-being and body image perception in various populations [51]. At the same time, high body exposure in training attire and during performances promotes increased attention to appearance [41], which may lead to the internalization of aesthetic norms promoted by the media and the sports community and increased pressure to achieve an athletic physique, increasing the risk of body dissatisfaction and unfavorable attitudes toward one’s appearance [52]. Consequently, pole dancing may impact body image both positively, by increasing satisfaction with body function and condition, and negatively, by increasing aesthetic pressure and internalizing appearance norms. These mechanisms may explain both the higher scores of women practicing pole dancing on the Internalization-Pressure and Internalization-Athlete scales, as well as the higher incidence of underweight, while simultaneously strengthening the functional component of self-esteem, especially in terms of physical condition.

Previous studies clearly indicate the prevalence of body dissatisfaction and eating disorders in athletes. Although these disorders are associated with poorer athletic performance and health, they are often overlooked by both the team and the athlete themselves, therefore further research is necessary in this area.

Due to the cross-sectional design of the study, causal relationships between sociocultural attitudes toward appearance and body image cannot be inferred. Longitudinal or experimental studies are necessary to determine the temporal and potentially causal nature of these associations.

### Strengths and Limitations

The study’s strengths include the inclusion of two groups of participants: women practicing pole dance and those who did not, which allowed for comparative analysis between the groups and the identification of potential differences. In addition to a proprietary survey questionnaire used to characterize the study group, the study utilized two standardized tools: the BES and the SATAQ 3. The use of these tools increases the reliability of the results, enables comparison with previous studies, and supports the reproducibility of the research for further analysis on this topic or in other sports. An additional advantage of the study is the inclusion of women practicing pole dance, which is significant given the limited number of studies on this discipline and the small sample sizes in the existing literature. The growing popularity of pole dance and the documented influence of media on body image and self-acceptance indicate the need for further research in this area.

The study also has limitations that may impact the interpretation of the results. The lack of a nationwide register of pole dance schools in Poland prevents appropriate sample selection, which may limit the representativeness of the results. Data collection via online surveys and the lack of data verification methods also limit the ability to fully verify their reliability, and weight and height were self-reported, which may be associated with measurement errors. Additionally, attention should be paid to the possibility of confounding factors, such as duration and intensity of training, socio-economic status or level of media exposure, which could have influenced the results. The use of non-probability purposive and convenience sampling increases the risk of selection bias and limits the generalizability of the findings to the broader population of women practicing pole dance in Poland. Furthermore, the study did not account for previous or current eating disorders, which could have influenced the responses. It should also be noted that the control group included women with varying levels of physical activity, which may have influenced the magnitude of the observed differences between groups. Therefore, further research is warranted, taking into account the identified factors, which could deepen understanding of the observed relationships and strengthen conclusions regarding the impact of this discipline on women’s physical and mental health.

## 5. Conclusions

Analysis of the results indicated that the majority of women practicing pole dancing were of normal body weight, although a small proportion were underweight. Compared with women not practicing pole dancing, obesity was less frequent in the pole dance group. Statistically significant differences in nutritional status were observed between the groups.

Women practicing pole dancing demonstrated more favorable self-evaluation only in the Physical Condition subscale, which includes aspects such as endurance, strength, and agility. They also presented higher levels of sociocultural attitudes toward appearance, particularly in terms of perceived media pressure and internalization of athletic body ideals. These differences reflect variations between groups rather than causal relationships.

Among women practicing pole dancing, a greater number of statistically significant associations were observed between body image and sociocultural attitudes compared to non-training women. Higher levels of perceived media pressure and internalization of appearance norms were correlated with lower body esteem in both groups.

Further analysis indicated that these associations were particularly evident among women with normal body weight. However, due to the cross-sectional design, the directionality of these relationships cannot be determined.

The findings emphasize that body image and sociocultural attitudes toward appearance are relevant factors in women practicing pole dance. These results may be useful for professionals such as dietitians, trainers, psychologists, and health educators when designing preventive and educational strategies aimed at promoting balanced and functional approaches to body perception. Given the limited research in this field, further longitudinal and experimental studies are warranted. As the study was conducted in Poland, cultural factors specific to this population may limit the generalizability of the findings to other sociocultural contexts.

## Figures and Tables

**Table 1:** BMI classification according to WHO [25].

BMI (kg/m^2^)	Interpretation of BMI
<18.5	Underweight
18.5–24.9	Body weight normal
25.0–29.9	Overweight
>30.0	Obesity

**Table 2:** Item assignment to the SATAQ 3 scales [27].

SATAQ 3 Scale	Item Numbers
Scale 1: Internalization -Pressure	2, 3, 6, 7, 9, 10, 13, 14, 15, 17, 20, 24
Scale 2: Internalization—Information Seeking	5(−), 8(−), 11(−), 12(−), 25(−), 26(−) *
Scale 3: Internalization—Athlete	18, 21, 22, 28
Scale 4: Information	1, 4, 16, 19, 23, 27

* (−) Items reverse-coded: 1 is scored as 5, 2 as 4, 3 as 3, 4 as 2, 5 as 1.

**Table 3:** Sten norms for women—SATAQ 3 [25].

Sten	Scale 1 Internalization-Pressure	Scale 2 Internalization-Information Seeking	Scale 3 Internalization-Athlete	Scale 4 Information
1	≤10	≤8	≤3	≤4
2	11–13	9–11	4–5	5
3	14–19	12–14	6–7	6–8
4	20–24	15–17	8–10	9–10
5	25–30	18–20	11–12	11–13
6	31–36	21–23	13–14	14–16
7	37–42	24–26	15–16	17–19
8	43–48	27–29	17–18	20–22
9	49–54	≥30	≥19	23–25
10	≥55			≥26

**Table 4:** Sten norms for women—interpretation of the BES [29].

Sten	Age (Years)																	
	16–19			20–29			30–39			40–49			50–59			≥60		
	SA	WC	PC	SA	WC	PC	SA	WC	PC	SA	WC	PC	SA	WC	PC	SA	WC	PC
1	≤34	≤13	≤22	≤37	≤16	≤23	≤35	≤18	≤22	≤33	≤16	≤20	≤31	≤15	≤18	≤26	≤10	≤12
2	35–37	14–18	23–24	38–40	17–21	24–25	36–38	19–21	23–25	34–37	17–20	21–23	32–35	16–19	19–21	27–31	11–15	13–16
3	38–41	19–22	25–27	41–43	21–25	26–28	39–42	22–25	26–27	38–40	21–24	24–26	36–38	20–22	22–24	32–35	16–20	17–21
4	42–44	23–26	28–30	44–47	26–29	29–31	43–45	26–29	28–30	41–44	25–28	27–29	39–42	23–26	25–27	36–40	21–24	22–25
5	45–48	27–31	31–32	48–50	30–33	32–34	46–49	30–33	31–32	45–47	29–32	30–32	43–46	27–29	28–30	41–44	25–29	26–29
6	49–51	32–35	33–35	51–53	34–37	35–36	50–52	34–37	33–35	48–51	33–36	33–34	47–49	30–33	31–33	45–49	30–34	30–33
7	52–55	36–40	36–37	54–56	38–42	37–39	53–55	38–41	36–38	52–55	37–40	35–37	50–53	34–37	34–36	50–53	35–39	34–37
8	56–58	41–44	38–40	57–60	43–46	40–42	56–59	42–45	39–40	56–58	41–44	38–40	54–56	38–40	37–39	54–57	40–44	38–41
9	59–61	45–49	41–43	61–63	47–50	43–44	60–62	46–48	41–43	59–62	45–48	41–43	57–60	41–44	40–42	58–62	45–48	42–45
10	≥62	≥50	≥44	≥64	≥51	≥45	≥63	≥49	≥44	≥63	≥49	≥44	≥61	≥45	≥43	≥63	≥49	≥46

SA—Sexual Attractiveness, WC—Weight Concern, PC—Physical Condition.

**Table 5:** Group Characteristics (*n* = 387).

	Group	X ± SD	Min	Max	*p*-Value	r
Age [years]	PDG (*n* = 207)	28.52 ± 6.37	16	55	0.199	0.07
	CG (*n* = 180)	31.18 ± 10.68	16	65		
Body mass [kg]	PDG (*n* = 207)	61.25 ± 9.27	42	110	<0.001 ***	0.23
	CG (*n* = 180)	66.76 ± 13.33	46	130		
Height [cm]	PDG (*n* = 207)	165.61 ± 6.30	150	181	0.582	0.03
	CG (*n* = 180)	165.77 ± 5.85	150	186		
BMI [kg/m^2^]	PDG (*n* = 207)	22.30 ± 2.84	16.33	36.33	<0.001 ***	0.22
	CG (*n* = 180)	24.27 ± 4.58	16.49	44.58		

X = average; SD = standard deviation; Min—minimum, Max—maximum; *** = *p* < 0.001.

**Table 6:** Training experience of women in the PDG (*n* = 207).

Training Experience	*n*	%
Up to 1 year	57	27.54
Over 1 year to 2 years	51	24.64
Over 2 years to 3 years	41	19.81
Over 3 years to 4 years	17	8.21
Over 4 years	41	19.81

**Table 7:** SATAQ 3 results summary (*n* = 387).

Scale	Study Group (*n* = 207) (X ± SD)	Control Group (*n* = 180) (X ± SD)	*p*-Value	r
Internalization—Pressure	35.35 ±13.25	31.54 ± 14.52	0.006 *	0.14
Internalization—Information Seeking	19.60 ± 4.92	18.89 ± 5.40	0.138	0.08
Internalization—Athlete	14.46 ± 3.96	11.13 ± 4.48	<0.001 ***	0.37
Information	14.15 ± 5.62	13.64 ± 6.54	0.225	0.06

X = average; SD = standard deviation; * = *p* < 0.05; *** = *p* < 0.001.

**Table 8:** BES results summary (*n* = 387).

Subsection	Study Group (*n* = 207) (X ± SD)	Control Group (*n* = 180) (X ± SD)	*p*-Value	r
Sexual Attractiveness	45.29 ± 8.50	44.47 ± 9.20	0.221	0.06
Weight Concern	32.10 ± 8.72	30.73 ± 9.88	0.145	0.07
Physical Condition	30.40 ± 7.17	28.83 ± 7.93	0.019 *	0.12

X = average; SD = standard deviation; * = *p* < 0.05.

**Table 9:** Statistical analysis of the relationships in groups between the sums of points obtained in the individual scales of the BES and the sums of points obtained in the individual scales of the SATAQ 3.

		SATAQ3			
BES	Group	Internalization–Pressure	Internalization–Information Seeking	Internalization–Athlete	Information
**Sexual Attractiveness**	PDG	−0.33 (*p* < 0.001 *)	−0.24 (*p* < 0.001 *)	−0.01 (*p* = 0.87)	−0.22 (*p* < 0.001 *)
	CG	−0.36 (*p* < 0.001 *)	−0.18 (*p* = 0.02 *)	−0.22 (*p* = 0.003 *)	−0.32 (*p* < 0.001 *)
**Weight Concern**	PDG	−0.51 (*p* < 0.001 *)	−0.34 (*p* < 0.001 *)	−0.19 (*p* = 0.01 *)	−0.35 (*p* < 0.001 *)
	CG	−0.31 (*p* < 0.001 *)	−0.12 (*p* = 0.10)	−0.14 (*p* = 0.06)	−0.19 (*p* = 0.01 *)
**Physical Condition**	PDG	−0.34 (*p* < 0.001 *)	−0.20 (*p* < 0.001 *)	−0.10 (*p* = 0.15)	−0.24 (*p* < 0.001 *)
	CG	−0.27 (*p* < 0.001 *)	−0.07 (*p* = 0.37)	−0.08 (*p* = 0.30)	−0.18 (*p* = 0.01 *)

* = *p* < 0.05.

**Table 10:** Statistical analysis of the relationships in groups between the sten scores awarded in the BES and the sten scores awarded in the SATAQ 3 scales.

		SATAQ3			
BES (STEN)	Group	Internalization–Pressure	Internalization–Information Seeking	Internalization–Athlete	Information
**Sexual Attractiveness**	PDG	−0.36 (*p* < 0.001 *)	−0.23 (*p* = 0.001 *)	−0.05 (*p* = 0.45)	−0.25 (*p* < 0.001 *)
	CG	−0.37 (*p* < 0.001 *)	−0.20 (*p* = 0.007 *)	−0.23 (*p* = 0.002 *)	−0.31 (*p* < 0.001 *)
**Weight Concern**	PDG	−0.49 (*p* < 0.001 *)	−0.31 (*p* < 0.001 *)	−0.21 (*p* = 0.002 *)	−0.33 (*p* < 0.001 *)
	CG	−0.29 (*p* < 0.001 *)	−0.12 (*p* = 0.10)	−0.14 (*p* = 0.055)	−0.14 (*p* = 0.054)
**Physical Condition**	PDG	−0.34 (*p* < 0.001 *)	−0.19 (*p* = 0.006 *)	−0.12 (*p* = 0.08)	−0.25 (*p* < 0.001 *)
	CG	−0.28 (*p* < 0.001 *)	−0.08 (*p* = 0.31)	−0.08 (*p* = 0.29)	−0.18 (*p* = 0.01 *)

* = *p* < 0.05.

## Data Availability

The data presented in this study are available on request from the corresponding author due to privacy restrictions.

## Abbreviations

The following abbreviations are used in this manuscript:

BES	Body Esteem Scale
BMI	Body Mass Index
CAWI	Computer-Assisted Web Interview
CBOS	Public Opinion Research Center (pol. Centrum Badania Opinii Społecznej)
CG	Control Group
max	Maximum
min	Minimum
PC	Physical Condition
PDG	Pole Dance Group
SA	Sexual Attractiveness
SATAQ 3	Sociocultural Attitudes Towards Appearance Questionnaire 3
SD	Standard Deviation
WC	Weight Concern
WHO	World Health Organization
X	Mean
